# Contrasting clonal and population genetic structure in two endangered Costa Rican *Vanilla* species of commercial interest

**DOI:** 10.1038/s41598-025-04101-5

**Published:** 2025-06-02

**Authors:** Maria Alejandra Serna-Sánchez, Adam P. Karremans, Diego Bogarín, Eric J. Fuchs

**Affiliations:** 1 Centro de Investigación Jardín Botánico Lankester, Universidad de Costa Rica, P.O. Box 302-7050, Cartago, Costa Rica; 2 Programa de Posgrado en Biología, Sistema de Estudios de Posgrado, Universidad de Costa Rica, San Pedro, San José 11501-2060 Costa Rica; 3 Escuela de Biología, Universidad de Costa Rica, Ciudad Universitaria Rodrigo Facio, San Pedro Montes de Oca, 11501-2060 Costa Rica; 4 Evolutionary Ecology Group, Naturalis Biodiversity Center, Leiden, The Netherlands; 5 Laboratorio Binacional, UNAM-UCR, Morelia, Michoacán Mexico

**Keywords:** Fine-scale genetic structure, Microsatellites, Pacific region, Population genetics, Ecology, Evolution, Genetics, Plant sciences

## Abstract

Wild relatives of the commercially grown *Vanilla planifolia*, such as *Vanilla odorata* and *V. pompona*, are understudied despite their ecological and agricultural importance. Both species are listed as endangered by the IUCN, but limited research on their genetic diversity hinders effective management. While *Vanilla* species reproduce extensively by cloning, wild populations may retain significant genetic diversity valuable for crop improvement and conservation. To evaluate genetic diversity and structure, we analyzed 146 individuals from 10 *V. pompona* populations and 75 individuals from six *V. odorata* populations in Costa Rica, using 11 and 10 microsatellites, respectively. *Vanilla odorata* showed higher clonality than *V. pompona* (63% vs. 35%). Both species exhibited low to moderate genetic diversity (*V. odorata*: H_O_ = 0.47, H_E_ = 0.29; *V. pompona*: H_O_ = 0.43, H_E_ = 0.41). *Vanilla odorata* populations revealed greater genetic differentiation than *V. pompona* populations (Nei’s G_ST_ = 0.51 vs. 0.091), with *V. odorata* also exhibiting isolation by distance (R² = 0.704, *p* < 0.05). Both species showed fine-scale genetic structure, with higher levels linked to clonality. Gene flow in *V. pompona* benefits from broad distribution and sexual reproduction, while *V. odorata* experiences limited gene flow due to its high clonality and restricted range. Protecting habitats and promoting outcrossing may aid *V. odorata* viability while *V. pompona* serves as an important resource for increasing genetic diversity in cultivated vanilla.

**Supplementary Information:**

The online version contains supplementary material available at 10.1038/s41598-025-04101-5.

## Introduction

Approximately 80 million years ago, the Vanilloideae subfamily diverged within Orchidaceae^1^, which includes the genus *Vanilla* Miller. This genus of culinary and cosmetic value includes around 140 species^2^. Among these, only *Vanilla planifolia* Andrews is cultivated on a large scale, with *Vanilla pompona* Schiede and the hybrid *Vanilla* × *tahitensis* (*V. planifolia* Andrews ~ *Vanilla odorata* C.Presl) cultivated to a lesser extent^3^. They are cultivated for their fruits rich in the organic compound vanillin^4,5^. *Vanilla planifolia* crops typically have low genetic diversity due to the use of a small number of clones for reproduction^6,7^, which can result in low resilience to environmental changes and biotic factors such as pests and pathogens^8^. Moreover, modern plantations rely on cultivated clones originally introduced from Mesoamerica to the rest of the world during the late 16th century^9^. Certain varieties, such as the high yielding ‘Mansa’ variety (with more than 75% of fruits retained), are commonly marketed for establishing new plantations, resulting in an industry that relies on a limited genetic base^10^.

Although commercially *V. planifolia* has been the most exploited species, the neotropics have around 41 aromatic *Vanilla* species with the potential for commercial use^11^. In Costa Rica, two wild species with fragrant fruits, *V. odorata* and *V. pompona*, represent critical genetic resources for *Vanilla* cultivation and could potentially contribute to the vanilla extract trade^2^, despite being classified as endangered by the International Union for Conservation of Nature (IUCN). Since 2004, studies on genetic diversity in some *Vanilla* species have been conducted using markers ranging from RAPDs to SNPs. Most of these studies have focused on species-level diversity, with few examining population-level genetic diversity and structure^3,6,8,9,12–17^. In these studies, *V. dilloniana*, *V. imperialis*, and *V. pompona* exhibited higher average heterozygosity compared to *V. planifolia*, *V. odorata*, and *V. × tahitensis*^*5,9*^. Despite efforts to advance our understanding of *Vanilla* genetics, a significant research gap remains concerning the clonal structure, population genetic diversity, and genetic structure of wild *V. odorata* and *V. pompona* populations. This knowledge gap, essential for effectively managing and conserving these species, will be addressed in this study.

Species with allogamous reproduction and the ability for long-distance seed dispersal are likely to exhibit higher levels of genetic variation and lower levels of population differentiation compared to species that regularly self-fertilize and have limited dispersal of propagules^18^. While certain species within *Vanilla* reproduce naturally via allogamous sexual reproduction or self-pollination, the overall success of natural pollination in *Vanilla* is low, as commonly observed in other food-deceptive orchids. For example, natural fruit set in *V. planifolia* in Puerto Rico and Central America is between 1 and 3%^19^, while Soto Arenas (1999) reported an even lower rate in Mexico: 1 fruit per 100–1000 flowers^22^. This is consistent with the 0.65% fertilization ratio reported by Pemberton et al. (2023) in southern Florida^23^ and the 4.9% found by Quezada-Euán et al. (2024) in Yucatán, Mexico^24^. Therefore, vegetative or clonal propagation is the most plausible and economically viable type of reproduction in the genus^25^. This may result in widespread spatial distribution of single genets. In natural environments, *Vanilla* individuals may expand over large areas; for example, an individual of *V. planifolia* was observed to cover areas up to 0.2 hectares^22^.

Although populations of *V. odorata* and *V. pompona* have been documented in Costa Rica, their genetic structure and diversity remain unknown. The primary objective of this project is to study the genetic diversity and clonal structure of *V. pompona* and *V. odorata* populations in Costa Rica using microsatellites. We hypothesize that *V. pompona* exhibits higher genetic diversity than *V. odorata* due to its wider distribution and larger population sizes. However, because *V. odorata* and *V. pompona* can reproduce clonally, we expect low overall genetic diversity, significant fine-scale genetic structure, and significant differences in allele frequencies among populations. Understanding the genetic diversity of these *Vanilla* species will provide baseline information for conservation strategies, such as increasing population connectivity or providing guidelines on best practices for translocating individuals among populations. It will also encourage the use of these species to improve the genetic resources of extensively cultivated *Vanilla*. A comprehensive understanding of the genetic diversity and reproductive biology of *Vanilla* species, including clonality and inbreeding in fragmented and geographically distant populations, is essential to preserve the genetic diversity of *Vanilla* crop wild relatives (CWRs) while simultaneously mitigating the impact of extreme climates on vanilla cultivation.

## Results

### Clonal assignment and estimates

Among the 75 *V. odorata* individuals collected in the Osa Peninsula, we identified 26 unique genets across all populations. *Vanilla odorata* has an average clonality of 63%. There is great variation in the level of clonal reproduction among populations, with Miramar and VT having the largest levels of clonality (75%). According to Simpson’s Diversity Index (SDI), the probability of finding two individuals with different genotypes is 25% in Miramar, while in Matapalo it rises to 80%. The average evenness is 0.64, indicating moderate genotypic diversity, as the distribution of genotypes within populations is not completely equitable, but neither is it extremely uneven. Matapalo exhibited the highest evenness (0.93), suggesting a homogeneous frequency distribution of all genotypes. In contrast, VT had the lowest evenness (0.43), indicating that a single genotype is predominant throughout the population (Table 1, Supplementary Fig. S1).

**Table 1:** Clonal diversity estimates for *V. odorata* populations in Southern Costa Rica.

Population	Number of individuals	Genets	Clonality (%)	Simpson’s Diversity Index (SDI)	Evenness
PST	24	8	66.66	0.76 (0.74–0.77)	0.46 (0.43–0.48)
VT	16	4	**75.00**	0.44 (0.74–0.77)	0.43 (0.38–0.50)
PPT	14	6	57.14	0.75 (0.71–0.78)	0.54 (0.49–0.60)
Miramar	8	2	**75.00**	0.25 (0.00-0.29)	0.64 (0.66-1.00)
Tigre	8	3	62.50	0.68 (0.57–0.71)	0.82 (0.78–0.98)
Matapalo	5	3	40.00	**0.80** (0.67–0.83)	**0.93** (0.89-1.00)
Mean	12.50 ± 2.84	4.33 ± 0.91	62.71 ± 5.36	0.61 ± 0.09	0.64 ± 0.08

Number of sampled individuals, number of unique genets, clonal percentage, Simpson’s diversity index, and evenness for each population, along with mean ± standard error values across all populations. Jackknife estimates for SDI and evenness are provided in parentheses.

Values ​​in bold indicate the highest within each metric, for comparative and visual interpretation purposes only.

Among the 146 *V. pompona* individuals collected in the Pacific Region of Costa Rica, we identified a total of 93 unique genets. *Vanilla pompona* has significantly less clonality, with an average clonality of 35%. Similar to *V. odorata*, there is significant variation in clonality across populations, with VT exhibiting the highest levels at 75%. According to the Simpson’s Diversity Index, the probability of finding two different genets in VT is 66%, while in Rio Jaris the probability of finding two individuals with different genets is 100%. Average evenness is 0.74, suggesting a balanced and diverse meta-population, with multiple genotypes present in relatively similar proportions (Table 2).

**Table 2:** Clonal diversity estimates for *Vanilla pompona* populations across the Pacific region of Costa Rica.

Population	Number of individuals	Genets	Clonality (%)	Simpson’s Diversity Index (SDI)	Evenness
STH	20	17	15.00	**0.98** (0.98–0.99)	**0.91** (0.90–0.92)
VT	20	5	**75.00**	0.66 (0.63–0.67)	0.54 (0.54–0.61)
PST	12	6	50.00	0.82 (0.78–0.86)	0.67 (0.61–0.75)
Los Chocuacos	16	9	43.75	0.87 (0.85–0.89)	0.59 (0.59–0.64)
Clavera	18	14	22.22	**0.97** (0.96–0.98)	0.83 (0.82–0.90)
Pavona	18	13	27.77	0.95 (0.94–0.96)	0.73 (0.72–0.82)
Garabito	8	4	50.00	0.82 (0.76–0.86)	0.89 (0.82–0.96)
Rio Jaris	8	8	0.00	**1.00** (1.00–1.00)	**1.00** (1.00–1.00)
San Rafael	18	12	33.33	0.92 (0.91–0.93)	0.64 (0.64–0.69)
El Hacha	8	5	37.50	0.79 (0.71–0.86)	0.64 (0.65–0.75)
Mean	14.60 ± 1.60	9.30 ± 1.41	35.45 ± 6.64	0.88 ± 0.03	0.74 ± 0.05

Number of sampled individuals, number of unique genets, clonal percentage, Simpson’s diversity index (SDI), and evenness for each population, along with mean ± standard error values across all populations. Jackknife estimates for SDI and evenness are provided in parentheses.

Values ​​in bold indicate the highest within each metric, for comparative and visual interpretation purposes only.

Furthermore, our results show that the average maximum clonal patch size in *V. odorata* was 36.2 (± 13.9) meters (Supplementary Fig. S1). For *V. pompona*, the average maximum spread of clones was 28.4 (± 12.5) meters (Supplementary Fig. S2). An intermixed pattern of genets was observed in both species, with different clones and genets coexisting in the same area. However, in *V. odorata* populations such as Miramar, VT, and PST, a few genets dominate, creating large clonal patches that reach up to 10 m in Miramar and at least 74 m in VT (Supplementary Fig. S1). A similar pattern was observed in *V. pompona*, although with greater clonal diversity (SDI) within populations. Only a few populations had large clones, such as VT, which had two genets that extended up to 32 m, and Los Chocuacos, where one genet extended up to 31 m. In the case of El Hacha, the same genet was identified in two individuals separated by up to 112 m.

### Genetic diversity

All markers in both species deviated from Hardy-Weinberg equilibrium (HWE), as expected for clonal species. Across the 10 loci analyzed in *V. odorata*, the number of alleles ranged from two (mVplCIR047) to six (mVhuCIR07). In *V. pompona*, the 11 loci analyzed exhibited a broader range, with three to 15 alleles, with mVhuCIR07 being the most polymorphic. Our estimates of genetic diversity were similar between populations within each species, with few differences between analyses including and excluding clones, except possibly in F_IS_. Genetic diversity estimates were slightly influenced by sample size. For example, in *V. odorata* in Miramar, we found the lowest diversity metrics because it is the population with the smallest sample size. The two populations with the fewest genets (VT and Garabito) also had the lowest genetic diversity estimates in *V. pompona*. *Vanilla odorata* shows lower diversity metrics than *V. pompona*, with fewer alleles (A = 1.70 vs. 2.76), lower allelic richness (Ar = 1.57 vs. 2.30), and a smaller effective number of alleles (Ae = 1.57 vs. 2.09). Although *V. pompona* has higher expected heterozygosity (He = 0.40, 95% CI = 0.20–0.58) compared to *V. odorata* (He = 0.27, 95% CI = 0.07–0.45), confidence intervals overlap. There is no clear geographic pattern in the distribution of genetic diversity. Inbreeding in *V. pompona* was low and not significant (F_IS_ = -0.03, SE = 0.19), while a significant heterozygote excess was found in *V. odorata* (F_IS_ = -0.67, SE = 0.17) (Tables 3 and 4).

**Table 3:** Genetic diversity estimates for six populations of *V. odorata* in Costa Rica, based on a dataset including clones (IC) and excluding clones (EC). *N* sample size, *A* average number of alleles per locus, *Ar* allelic richness, *Ae* effective number of alleles, *Ho* observed heterozygosity, *He* expected heterozygosity, *F*_*IS*_ inbreeding coefficient. Mean values ± (SE). *Indicates significant F_IS_ (*P* < 0.01) based on 1000 bootstrap replicates.

Population	Dataset	*N*	A	Ar	Ae	Ho	He	F_IS_
PST	IC	24	1.8 (0.25)	1.72 (0.21)	1.70 (0.21)	0.55 (0.15)	0.32 (0.09)	-0.76* (0.14)
	EC	8	1.8 (0.25)	1.62 (0.18)	1.67 (0.20)	0.52 (0.15)	0.32 (0.09)	-0.68* (0.14)
VT	IC	16	1.9 (0.23)	1.76 (0.21)	1.71 (0.21)	0.58 (0.16)	0.33 (0.09)	-0.71* (0.14)
	EC	4	1.9 (0.23)	1.68 (0.16)	1.64 (0.17)	0.55 (0.15)	0.34 (0.08)	-0.59* (0.19)
PPT	IC	14	1.9 (0.23)	1.79 (0.22)	1.73 (0.22)	0.57 (0.15)	0.33 (0.09)	-0.67* (0.14)
	EC	6	1.9 (0.23)	1.68 (0.18)	1.73 (0.21)	0.57 (0.15)	0.35 (0.09)	-0.62* (0.15)
Miramar	IC	8	1.4 (0.16)	1.36 (0.15)	1.31 (0.15)	0.31 (0.15)	0.16 (0.07)	-0.75* (0.16)
	EC	2	1.4 (0.16)	1.40 (0.16)	1.36 (0.15)	0.35 (0.15)	0.20 (0.08)	-0.75* (0.16)
Tigre	IC	8	1.5 (0.22)	1.48 (0.21)	1.39 (0.16)	0.34 (0.15)	0.20 (0.08)	-0.68 (0.20)
	EC	3	1.5 (0.22)	1.46 (0.20)	1.46 (0.19)	0.33 (0.15)	0.23 (0.10)	-0.60 (0.25)
Matapalo	IC	5	1.6 (0.22)	1.60 (0.22)	1.56 (0.20)	0.48 (0.16)	0.26 (0.09)	-0.83* (0.07)
	EC	3	1.6 (0.22)	1.55 (0.19)	1.54 (0.19)	0.47 (0.16)	0.27 (0.09)	-0.77* (0.10)
Mean	IC	75	1.7 (0.22)	1.62 (0.20)	1.57 (0.19)	0.47 (0.15)	0.27 (0.09)	-0.73* (0.14)
	EC	26	1.7 (0.22)	1.57 (0.18)	1.57 (0.19)	0.47 (0.15)	0.29 (0.09)	-0.67* (0.17)

**Table 4:** Genetic diversity estimates for 10 populations of *V. pompona* in Costa Rica, based on a dataset including clones (IC) and excluding clones (EC). *N* sample size, *A* average number of alleles per locus, *Ar* allelic richness, *Ae* effective number of alleles, *Ho* observed heterozygosity, *He* expected heterozygosity, *F*_*IS*_ inbreeding coefficient. Mean values ± (SE). *Indicates significant F_IS_ (*P* < 0.01) based on 1000 bootstrap replicates.

Population	Dataset	*N*	A	Ar	Ae	Ho	He	F_IS_
STH	IC	20	2.91 (0.64)	2.48 (0.43)	2.03 (0.35)	0.44 (0.12)	0.40 (0.08)	-0.05 (0.16)
	EC	17	2.91 (0.64)	2.26 (0.34)	2.10 (0.38)	0.44 (0.12)	0.42 (0.08)	-0.04 (0.16)
VT	IC	20	2.09 (0.28)	1.99 (0.27)	1.73 (0.28)	0.40 (0.12)	0.31 (0.08)	-0.24 (0.14)
	EC	5	1.91 (0.28)	1.86 (0.27)	1.59 (0.22)	0.33 (0.12)	0.30 (0.08)	-0.06 (0.19)
PST	IC	12	2.55 (0.34)	2.24 (0.26)	1.75 (0.19)	0.55 (0.13)	0.36 (0.07)	-0.37* (0.16)
	EC	6	2.45 (0.37)	2.20 (0.29)	1.85 (0.23)	0.52 (0.13)	0.39 (0.08)	-0.28* (0.15)
Los Chocuacos	IC	16	2.82 (0.6)	2.46 (0.41)	2.16 (0.35)	0.53 (0.14)	0.46 (0.07)	-0.12 (0.23)
	EC	9	2.82 (0.60)	2.35 (0.36)	2.21 (0.42)	0.50 (0.12)	0.46 (0.07)	-0.06 (0.22)
Clavera	IC	18	3.45 (0.78)	3.00 (0.60)	2.54 (0.67)	0.47 (0.12)	0.46 (0.08)	0.14 (0.20)
	EC	14	3.45 (0.78)	2.58 (0.41)	2.48 (0.61)	0.48 (0.12)	0.47 (0.07)	0.13 (0.21)
Pavona	IC	18	3.82 (0.84)	3.06 (0.55)	2.52 (0.53)	0.47 (0.11)	0.48 (0.08)	0.05 (0.13)
	EC	13	3.73 (0.84)	2.69 (0.40)	2.55 (0.52)	0.43 (0.11)	0.50 (0.08)	0.16 (0.15)
Garabito	IC	8	2.18 (0.44)	2.17 (0.44)	1.97 (0.40)	0.35 (0.14)	0.36 (0.10)	-0.04 (0.25)
	EC	4	2.18 (0.44)	2.18 (0.44)	1.94 (0.38)	0.36 (0.14)	0.39 (0.10)	-0.01 (0.25)
Rio Jaris	NC	8	2.73 (0.69)	2.26 (0.45)	2.12 (0.48)	0.43 (0.13)	0.37 (0.10)	-0.11 (0.19)
San Rafael	IC	18	2.82 (0.55)	2.42 (0.42)	1.90 (0.33)	0.35 (0.12)	0.35 (0.09)	0.14 (0.18)
	EC	12	2.82 (0.55)	2.21 (0.33)	1.89 (0.29)	0.37 (0.12)	0.38 (0.08)	0.10 (0.18)
El Hacha	IC	8	2.55 (0.43)	2.50 (0.42)	1.99 (0.27)	0.45 (0.13)	0.40 (0.10)	-0.14 (0.17)
	EC	5	2.55 (0.43)	2.42 (0.39)	2.12 (0.33)	0.45 (0.13)	0.43 (0.11)	-0.11 (0.16)
Mean	IC	146	2.79 (0.56)	2.50 (0.44)	2.07 (0.39)	0.44 (0.13)	0.40 (0.09)	-0.07 (0.18)
	EC	93	2.76 (0.56)	2.30 (0.37)	2.09 (0.39)	0.43 (0.12)	0.41 (0.09)	-0.03 (0.19)

### Genetic structure and isolation by distance

**Fig. 1:**
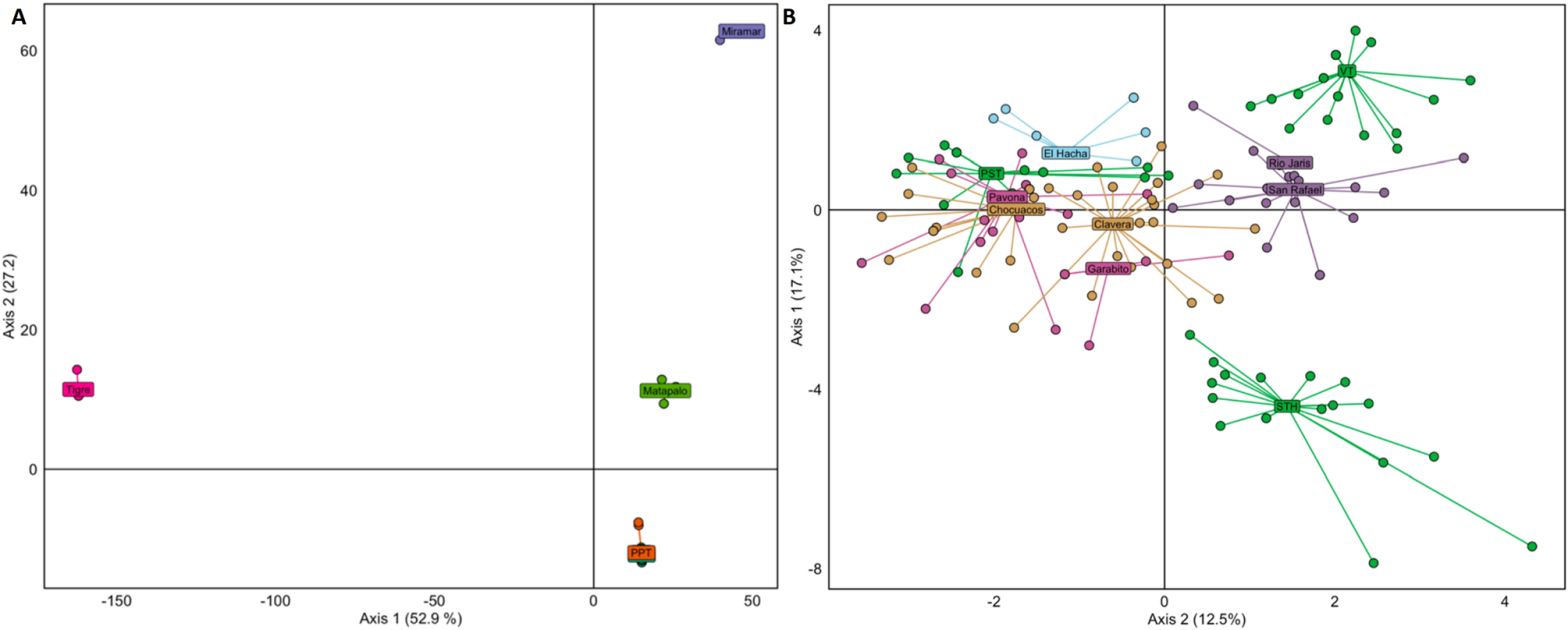
Discriminant analysis of principal components (DAPC) **(A)** clustering of six populations of *V. odorata*, including clones (IC). **(B)** clustering of ten populations in *V. pompona*, including clones. Colors denote the geographical allocation of *V. pompona* populations (clusters) to designated regions: Green = South Pacific - Peninsula; Yellow = South Pacific - Térraba; Pink = Central Pacific - Carara; Blue = North Pacific; Purple = Central Pacific - Puriscal.

We found significant genetic structure at the population level in both species: G_ST_ = 0.514 (95% CI = 0.500–0.529) for *V. odorata*; and G_ST_ = 0.091 (95% CI = 0.075–0.108) for *V. pompona*. Structure was significantly higher in *V. odorata*. The DAPC analysis of *V. odorata* shows a clear separation of populations in the inner part of the peninsula and those populations in Piro, forming a separate cluster (Fig. 1A). We found significant IBD for *V. odorata* (r_IC_ = 0.704, *p* < 0.05; r_EC_ = 0.737, *p* < 0.05; Supplementary Fig. S3).

In *V. pompona*, the DAPC analysis revealed a very low level of genetic structure, resulting in most populations clustering together. Only two populations, STH and VT, stand out as genetically distinct. Populations from the South Pacific–Térraba (Clavera and Los Chocuacos), Central Pacific–Carara (Pavona and Garabito), North Pacific (El Hacha), and PST all form a central cluster, reflecting similar allele frequencies (Fig. 1B, Supplementary Fig. S6). Additionally, San Rafael and Rio Jaris form a separate but closely related cluster to the main group (Fig. 1B, Supplementary Fig. S6). No significant IBD was found among *V. pompona* populations (r_IC_ = 0.138, *p* = 0.208; r_EC_ = 0.056, *p* = 0.275; Supplementary Fig. S4) or at the regional level (r_IC_ = 0.351, *p* = 0.187; r_EC_ = 0.533, *p* = 0.123; Supplementary Fig. S5).

### Fine scale spatial genetic structure

**Fig. 2:**
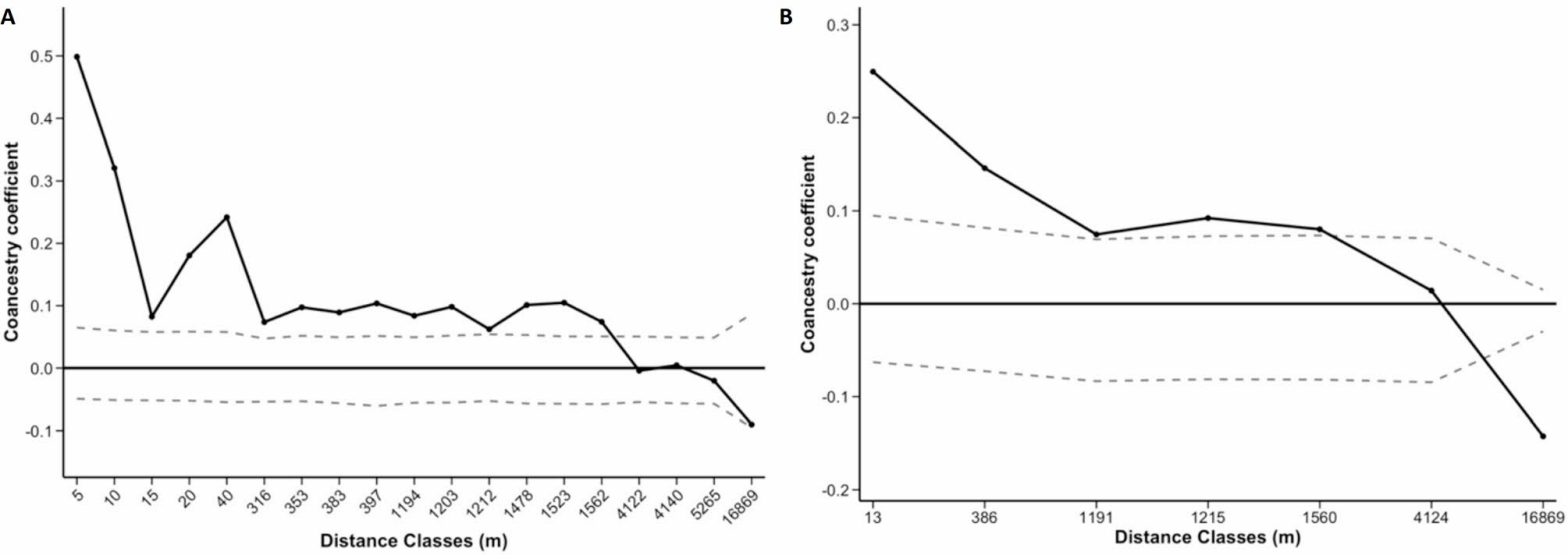
Correlograms showing the spatial autocorrelation of the coancestry Loiselle coefficient (Loiselle et al., 1995) as a function of distance for *V. odorata* populations: **(A)** dataset including clones, **(B)** dataset excluding clones. Dashed lines represent the lower and upper 95% confidence limits for the null hypothesis of no spatial genetic structure.

**Fig. 3:**
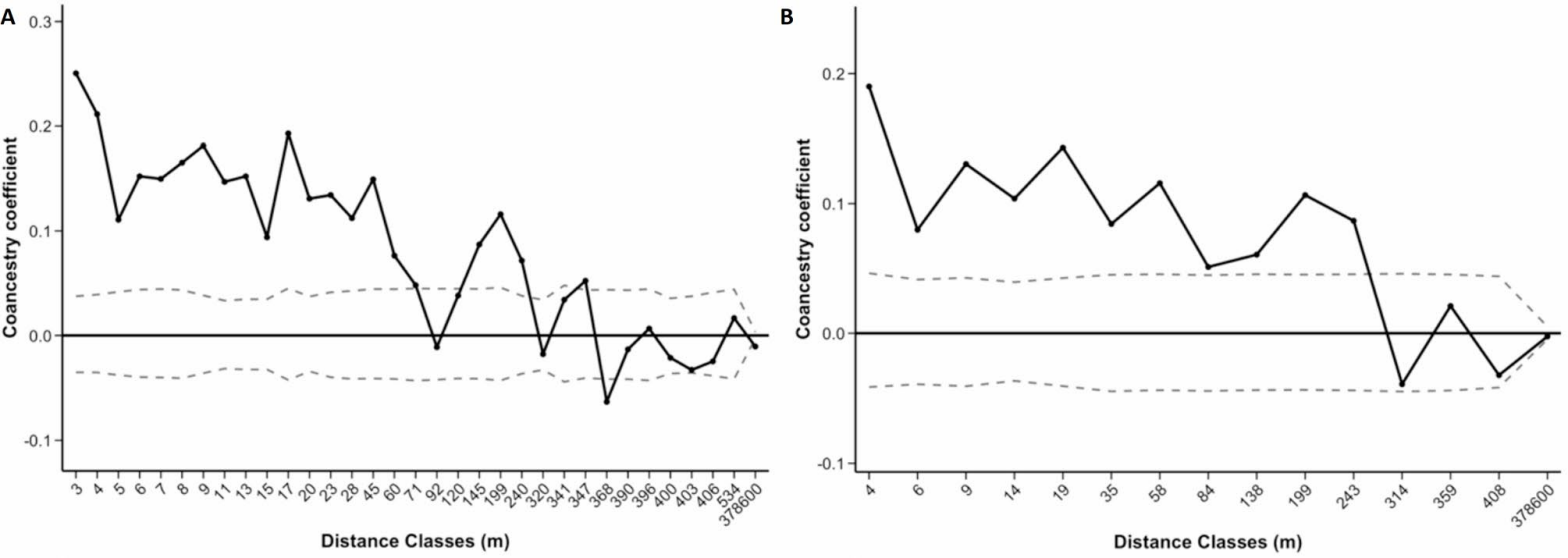
Correlograms showing the spatial autocorrelation of the coancestry Loiselle coefficient (Loiselle et al., 1995) as a function of distance for *V. pompona* populations: **(A)** dataset including clones, **(B)** dataset excluding clones. Dashed lines represent the lower and upper 95% confidence limits for the null hypothesis of no spatial genetic structure.

For both species, pairwise relatedness decreased with increasing pairwise distances between individuals (Figs. 2 and 3). The point at which the correlogram crossed the X-axis allowed us to calculate the average patch size^26,27^. In *V. odorata*, the average patch size was estimated at around 4120 m for both datasets (Fig. 2). For *V. pompona*, average patch size was approximately 92 m when clones were included and 283 m when clones were excluded (Fig. 3). Autocorrelation between *V. odorata* individuals separated by less than 5 m, including ramets (*r* = 0.498; Fig. 2A), was significantly different from zero and twice as large as for our data set with only genets (*r* = 0.249; Fig. 2B). In *V. pompona*, autocorrelation between individuals separated by less than 3 m, including ramets (*r* = 0.250; Fig. 3A), was significant and slightly higher than for individuals separated by less than 4 m in the genets subset (*r* = 0.190; Fig. 3B). However, spatial autocorrelation remains significant and high in *V. odorata* up to 1560 m, and up to 60 m and 243 m in *V. pompona* when clones are included (IC) and excluded (EC), respectively (Figs. 2 and 3). Loiselle’s coancestry coefficient was consistently higher for the ramet dataset than for the genet dataset, indicating a strong influence of clones in shaping spatial genetic structure in the closest distance classes. When FSGS analyses were performed by population for each species, similar patterns were observed (Supplementary Figs. S7 and S8). However, individual autocorrelation was lower because larger distance classes were needed due to the limited number of individuals within populations.

## Discussion

In our studies, the clonal nature of *V. odorata* and *V. pompona* has proven to be a critical factor in understanding the amount and distribution of genetic diversity. In comparing *V. odorata* and *V. pompona*, our study highlights sharp differences in clonality, geographic distribution, genetic diversity, and structure. In Costa Rica, while *V. odorata* is more clonal and has a restricted distribution, primarily confined to the Osa Peninsula, *V. pompona* has a broader distribution along Costa Rica’s Pacific coast. As expected, *V. pompona* showed higher levels of genetic diversity compared to *V. odorata*, likely due to its wider distribution, reduced clonality, and increased opportunities for sexual reproduction and gene flow. This is confirmed by *V. pompona*’s lack of inbreeding and low levels of spatial genetic structure in contrast with *V. odorata*. The restricted and clonal nature of *V. odorata* populations is consistent with our predictions, as it results in heterozygote excess due to its limited genetic diversity and predominantly vegetative propagation.

In our results, repeated genotypes were considered products of vegetative reproduction, particularly guerrilla-type growth, where ramets are widely spaced and rapidly spread into new areas^28^. Vegetative or clonal reproduction in orchids often correlates with reduced genetic diversity, as observed in *Vanilla planifolia*^6,7^. Similarly, the orchid *Pelatantheria scolopendrifolia* (Makino) Aver. had moderate to low levels of genetic diversity, attributed primarily to small population sizes, demographic events, clonal reproduction, and limited gene flow^29^. Clonal architecture, which influences the spatial distribution of ramets, can significantly impact geitonogamy^30^. In populations of *V. odorata*, such as Miramar, Tigre, VT, and PST, a clumped distribution of ramets was observed. In *V. odorata*, this clustering will likely lead to a higher degree of geitonogamy^30^ and may contribute to the significant genetic structure observed among populations. Conversely, *V. pompona* individuals are typically more intermingled (except in VT), which may lead to higher outcrossing rates^30^. A mixed distribution of genets can improve outcrossing by increasing the availability of diverse pollen from multiple genets^30^. This pattern is commonly observed in populations of *V. pompona* (Supplementary Fig. S2).

The near-zero inbreeding coefficient observed in *V. pompona* (F_IS_ = -0.03) is in direct contrast with high inbreeding values reported for *V. mexicana* (F_IS_ = 0.74), where autogamy is prevalent^16^. Thus, the higher levels of genetic diversity observed in *V. pompona* are most likely due to relatively higher levels of sexual reproduction compared to clonal growth. In contrast, *V. odorata’s* heterozygote excess (F_IS_ = -0.67) may be attributed to its high clonal reproduction rates that maintain large fractions of heterozygous ramets within populations. This pattern is also consistent with bottleneck or founder effects, where guerrilla propagation of clones may lead to the widespread establishment of heterozygous genotypes^31^. Clonal reproduction may maintain heterozygosity or even increase it by mutation over generations^32,33^.

The genetic diversity observed in *Vanilla odorata* and *V. pompona* aligns with patterns observed in other orchids, with diversity levels influenced by the degree of clonality. In *V. odorata*, smaller and highly clonal populations, such as Miramar, Tigre, and Matapalo, showed notably lower heterozygosity values compared to larger, less clonal populations like PPT and PST. Similarly, in *V. pompona*, the more clonal populations VT, PST, and Garabito had reduced genetic diversity, whereas larger populations like STH, Clavera, and La Pavona exhibited higher genetic diversity. Previous studies have shown that in clonal orchids, such as *Cypripedium calceolus* L., populations that rely more heavily on clonal reproduction tend to have lower genetic diversity due to limited genetic recombination compared to populations that favor sexual reproduction^34^. When compared with other *Vanilla* species, *V. odorata* exhibits low to moderate genetic diversity, which aligns with findings for *V. planifolia*, another highly clonal species with a deceptive pollination system^21^. *Vanilla pompona* has higher levels of genetic diversity, similar to African *Vanilla* species (*V. madagascariensis*, *V. decaryana*, *V. humblotii*, *V. perrieri*, and *V. bosseri*), which combine both clonal and sexual reproductive strategies but tend to have lower clonal reproduction^15,35^. Like *V. pompona* populations in Costa Rica, *V. madagascariensis*, *V. decaryana*, and *V. perrieri* also tend to have larger populations and broader distributions across Madagascar, contributing to their higher genetic diversity. Together, these results underscore how reproductive strategy and population size can influence genetic diversity, with clonality often associated with reduced diversity due to limited genetic recombination and smaller effective population sizes^36,37^.

Gene flow within Orchidaceae has been shown to occur over distances of up to 250 km^38^, but greater population differentiation can arise when intermediate populations are scarce, limiting gene flow between distant populations^38^. The genetic structure in *Vanilla odorata* and *V. pompona* likely reflects the combined influence of limited gene flow through pollen and seed dispersal, shaped by distinct pollination and dispersal mechanisms in each species. In *V. odorata*, a higher population structure is observed, likely due to pollination through food deception by both male and female *Euglossa* bees^21^. In deceptive systems, pollinators receive no reward (such as food or fragrance), so they quickly learn to avoid these flowers, resulting in less frequent visits and, consequently, fewer opportunities for effective pollination^39^. Limited pollen dispersal in *V. odorata*, combined with short-range seed dispersal by fragrance and pulp collection by *Euglossa* bees and *Trigona fulviventris* (Meliponini)^40^, likely contributes to the Isolation by Distance (IBD) pattern and strong genetic clustering, particularly in geographically isolated populations like Tigre, Miramar, and Matapalo.

Conversely, *V. pompona*, which is distributed along the Pacific coast, exhibits lower genetic structure despite its broad range. This pattern can be explained by the stepping-stone theory, where populations connected by intermediate groups maintain genetic connectivity across larger distances, even if direct gene flow between distant populations is limited^41,42^. In *V. pompona*, *Eulaema cingulata* was identified as the main pollinator, engaging in floral scent collection and nectar searching^43^. This behavior facilitates pollen removal and attracts bees from long distances, potentially increasing gene flow^43^. In addition, *V. pompona* benefits from effective seed dispersers like the spiny rat (*Proechimys semispinosus*) and agouti (*Dasyprocta punctata*), which have extensive home ranges (up to 1,258 m during the rainy season) and play a significant role in maintaining genetic connectivity across Central American forests^44^. These dispersers help spread seeds over greater distances, supporting high genetic connectivity in *V. pompona* despite geographical separation. Overall, these findings highlight how biological interactions, as well as clonality, population size and gene flow, influence genetic structure in *V. odorata* and *V. pompona*.

Interspecific gene flow via hybridization may also contribute to genetic diversity and structure estimates in our samples, as *V. pompona* and *V. odorata* are sympatric. Although natural hybrids between *V. pompona* and *V. odorata* may exist, their occurrence under natural conditions is highly unlikely due to the specificity in plant-pollinator relationships and the specialized pollination strategies in *Vanilla*. As previously stated, the pollinators of *V. pompona* are male *Eulaema*, a large bee that is attracted to the flowers by floral fragrances, which are the main reward^43^. Conversely, the deceptive *V. odorata* is likely pollinated by smaller *Euglossa* or Meliponini bees, analogous to the pollination of the florally similar *V. planifolia*^21^. Furthermore, in natural conditions, interspecific pollen transfer is highly unlikely due to the lack of flowering overlap between both species^43,45^. Considering the morphological differences of pollinators and the lack of synchrony in flowering phenology, natural hybridization between *V. odorata* and *V. pompona* is unlikely.

Fine-scale genetic structure (FSGS) in plant populations is defined as the non-random distribution of genotypes at a local scale, which can be caused by limited seed dispersal, clonality, or environmental factors^46^. In *V. odorata*, the strong genetic structuring observed when clones were included, suggests that clonality plays a dominant role in shaping local genetic patterns, as reported in previous studies^14,36^. However, after removing clones, the genetic structure revealed a pattern consistent with full-sibling relationships^47^, indicating that seed dispersal in sexually reproduced progeny is highly localized. In *V. pompona*, a similar but less clear pattern was observed. The moderate relatedness observed when clones were included, indicates a mixture of clonal individuals, sexually produced progenies, and unrelated individuals. Once clones were excluded, relatedness decreased significantly but remained significant up to 240 m, indicating large clusters of related individuals composed of full- and half-siblings within *V. pompona* populations. A similar pattern was observed in *V. humblotii* Rchb.f., where FSGS was greater for the ramets (datasets with clones) than for the genets alone, suggesting that, like in *V. odorata* and *V. pompona*, the repeated multilocus genotypes (clones) play a significant role in defining a strong spatial genetic structure, particularly in the first distance classes^14^.

Despite *V. pompona*’s potential for long-distance gene dispersal, many seeds may remain in close proximity, leading to local FSGS (see Fig. 3). Studies regarding the seed dispersal of Neotropical *Vanilla* suggest, that although potentially being able to have long-distance dispersal, most seeds are likely being dispersed relatively short distances from the plant^40,48^. Specifically, *Vanilla pompona* fruits are indehiscent, naturally dropping below the plant when mature. The fruit is then consumed fully or in part by mammals, especially rodents, which can disperse the seeds up to 18 h after consumption, usually within its home range^40^. In the case of *Vanilla odorata* fruits are dehiscent, naturally exposing the seeds that are displaced by fragrance collecting male orchid bees, causing seeds to drop directly below the fruit^40^. This localized seed dispersal, coupled with the behavior of seed vectors, reinforces the development of fine-scale genetic structure in both *V. pompona* and *V. odorata*.

Our findings highlight that both population size and clonality significantly shape genetic diversity in *V. odorata* and *V. pompona*. Additionally, clonality increases genetic structuring, particularly in small, isolated populations. From a conservation perspective, our study underscores the importance of maintaining larger population sizes and reducing isolation to support genetic diversity, especially in clonal species like *V. odorata*, where genetic diversity is already limited. Preserving diverse and interconnected habitats for *V. pompona* could enhance gene flow, helping to maintain population sizes and resilience. For *V. odorata*, focusing on reducing genetic bottlenecks by protecting diverse habitats and promoting occasional outcrossing could mitigate genetic drift and support long-term viability. These strategies are essential for conserving these unique genetic resources and the adaptive potential of both species in Costa Rica’s tropical forests. To achieve this goal, integrated conservation strategies recommended by Watteyn et al. (2020) include protecting forest areas where wild vanilla species naturally occur, promoting reforestation of biological corridors, cultivating vanilla alongside native tree species with economic benefits, and implementing payments for ecosystem services to incentivize farmers and landowners to conserve native vanilla populations while adopting sustainable agricultural practices^11^.

Moreover, our results suggest that *V. pompona* may be an important resource to enhance the genetic diversity of cultivated vanilla through breeding strategies that include hybridization with *V. planifolia*. This approach could promote sexual reproduction while maintaining high genetic diversity. The broad distribution and reduced genetic structure of *V. pompona* make it a valuable resource from which individuals can be selected from various sites. Preliminary studies indicate that wild *V. pompona* populations possess desirable traits for commercial plantations, such as increased resistance to pathogens like *Fusarium oxysporum* f. sp. *vanillae* and increased drought tolerance, compared to cultivated *V. planifolia*^49,50^. Menchaca et al. (2011) confirmed the feasibility of generating hybrids between *V. planifolia* and *V. pompona*, which exhibited high germination rates and remarkable hybrid vigor^51^. On the other hand, it is crucial to note that when collecting individuals of *V. odorata* from patches of 30–40 m, there is a high likelihood that they belong to the same clone. This highlights the importance of considering spatial genetic structure when collecting and using wild populations for breeding programs. These findings emphasize the importance of conserving multiple populations to preserve the overall genetic diversity of the species.

While this study employed microsatellites and included a robust sampling effort, certain limitations should be acknowledged. The limited number of populations and markers, particularly for *Vanilla odorata*, a rare species confined to just a few localities in Costa Rica, may influence the scope of our findings. For future research, we recommend expanding marker types and genomic coverage, such as using SNPs, as well as incorporating nuclear and organellar markers like plastomes to provide complementary insights into the species’ evolutionary history. Additionally, including more populations across a broader geographic range and integrating landscape genetics would help to further explore how geographic and environmental factors shape genetic variation.

### Methods

#### Study species

*Vanilla* is a pantropical genus consisting of around 140 species of hemiepiphytic and epiphytic vines within the Orchidaceae family^2^. *Vanilla odorata* and *V. pompona* are distributed from Mexico to Peru and Brazil^45^, with both species present in Costa Rica’s Osa Conservation Area (ACOSA)^11^. *Vanilla pompona* also occurs along the Pacific slope of Costa Rica. Their flowers consist of three sepals, two lateral petals, and a large trumpet-shaped petal known as the labellum, which is partially fused to the column. This leads to a single ventral stigma, separated by the rostellum from the anther, which holds two pollen masses^45^. *Vanilla pompona* blooms in Costa Rica from January to February^43^, with flowers measuring 6 to 8 cm in length and 7.5 to 10 cm in diameter. The successive inflorescences have one to two open flowers at a time, which are ephemeral, lasting only one day and wilting after noon; they are pale yellow with a striking yellow-orange labellum and a strong mint-like fragrance^45^. In contrast, *Vanilla odorata* flowers bloom in April and May^45^, and are 8 cm long and 7 cm wide^45^. They feature successive inflorescences with one to two open flowers at a time, ephemeral from 7:00 a.m. to 4:00 p.m., with pale greenish-white tepals and a greenish-white labellum, and have a mild, fresh fragrance^45^.

### Sample collection

**Fig. 4:**
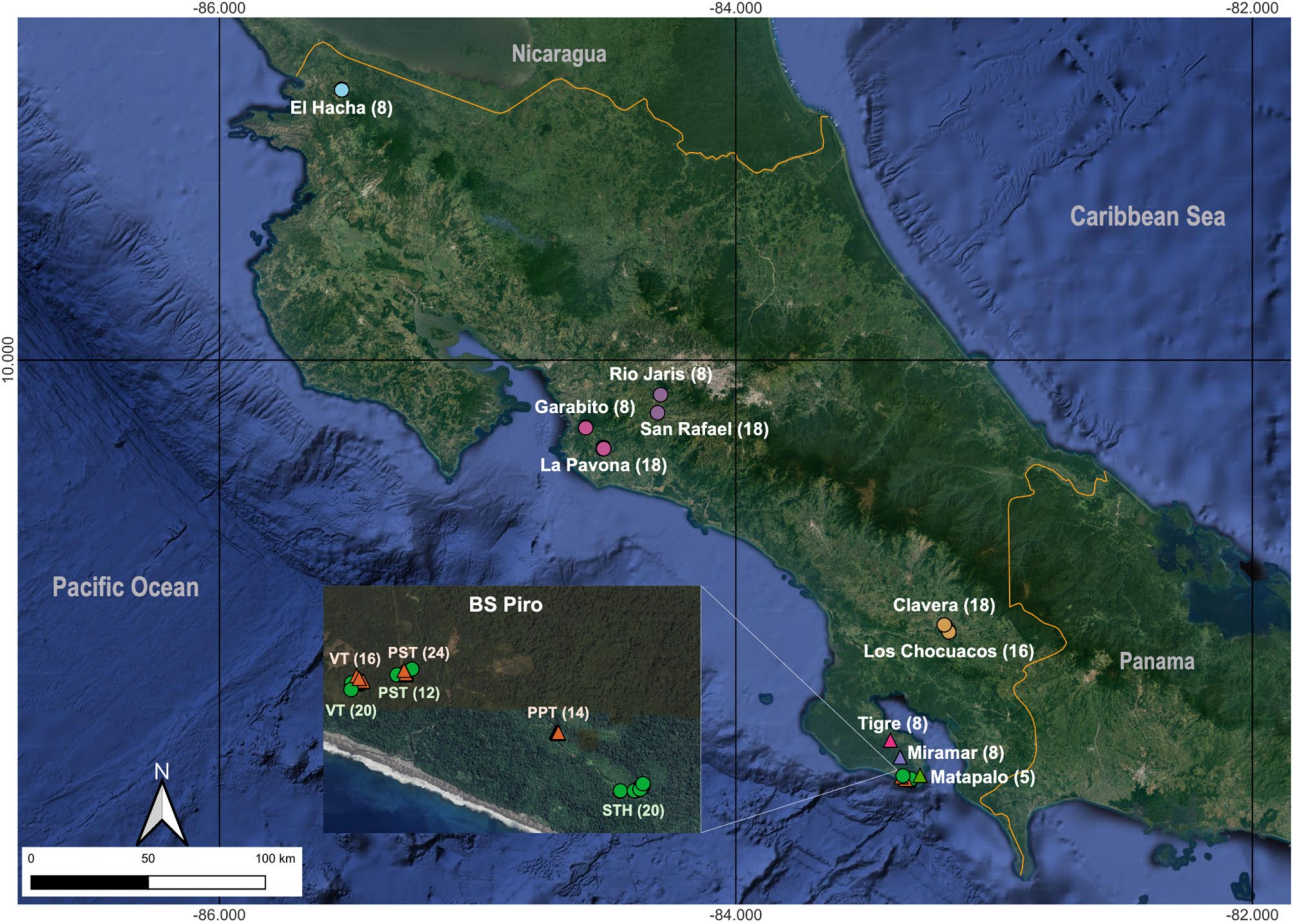
A map of Costa Rica showing the locations of *V. odorata* (triangles) and *V. pompona* (circles) populations. The colors grouping the circles in *V. pompona* correspond to the five regions in which the populations are categorized: Blue = North Pacific; Pink = Central Pacific - Carara; Purple = Central Pacific - Puriscal, Yellow = South Pacific - Térraba Green = South Pacific - Peninsula. The numbers in brackets indicate the number of individuals collected at each site. In the zoomed-in view of the Piro Biological Station, the names in light orange represent *V. odorata* populations, while the names in light green represent *V. pompona* populations. Abbreviations are as follows: VT = Vanilla Trail, PST = Piro Station Trail, PPT = Pica Pica Trail, STH = Stairway to Heaven. The map was created using QGIS software (version 3.30.3-‘s-Hertogenbosch, available at https://qgis.org/), with minor modifications made in Adobe Photoshop^®^ CC (Adobe Systems Inc., California, U.S.A.). QGIS is licensed under the GNU General Public License (https://www.gnu.org/licenses).

The localities cited by Karremans et al. (2020) for *V. odorata* and *V. pompona* were visited and sampled in the current study, with the materials collected being highly representative of the known populations of both species in the country. *Vanilla* plants used for this study were identified by A.P. Karremans, following the circumscription of Karremans et al. (2020). Representative vouchers for each study site for both species are kept as living plants or liquid specimens at Centro de Investigación Jardín Botánico Lankester (JBL), in Cartago, Costa Rica. A complete list of voucher numbers is provided in Supplementary Table S1. We selected 10 *V. pompona* populations along the Pacific slope of Costa Rica (Fig. 4). For *V. odorata*, we collected samples from six populations located in the Osa Peninsula (Fig. 4). We sampled between 8 and 20 individuals from each of the 10 *V. pompona* populations, totaling 146 individuals; these individuals were grouped into five regions (See Fig. 1). For *V. odorata*, we sampled from 5 to 24 individuals from each of the six populations. Each individual was georeferenced, and we collected one to two leaves per individual, which were stored in silica gel and processed within three days at the Laboratorio de Ecología Molecular of the School of Biology at Universidad de Costa Rica. All procedures complied with Costa Rican laws, and research permits were approved by the Research Committee of the School of Biology at the University of Costa Rica under project approval code C0049 from the Vice-Rectorate of Research.

### DNA extraction and SSR amplification

We extracted total DNA from leaf tissue using the modified CTAB protocol from Doyle and Doyle (1990)^52^. Dilutions were prepared to achieve a final concentration of 20 ng/µL for PCR reactions. Microsatellites (SSRs) previously proposed in the literature for other *Vanilla* species were used in this study^6,17^. A total of 28 SSR markers were tested for transferability, of which 16 successfully amplified in *V. pompona* and 15 in *V. odorata*. Among these, 12 markers were polymorphic for each species. However, one marker was excluded from the analysis in *V. pompona* and two in *V. odorata* due to missing data. Therefore, eleven microsatellite loci were used for *V. pompona*: mVhuCIR07, mVhuCIR08, mVhuCIR10, mVhuCIR11, mVroCIR01, mVroCIR05; mVplCIR015, mVplCIR016, mVplCIR019, mVplCIR026, mVplCIR031; while 10 microsatellite loci were used for *V. odorata*: mVhuCIR07, mVhuCIR08, mVroCIR01, mVroCIR03, mVroCIR05; mVplCIR05, mVplCIR025, mVplCIR026, mVplCIR031, mVplCIR047. The forward primer of each primer pair was fluorescently labeled at the 5’ end. PCR thermal profiles and primer mixes are described in Supplementary Table S2 of the supplementary material. We used a Veriti™ thermal cycler (Applied Biosystems, Foster City, CA, USA) to perform the PCR reaction. Amplification products were visualized in an ABI-3500 Sanger sequencer at Escuela de Biología at Universidad de Costa Rica, using Hi-Di™ Formamide and GeneScan™ 500 LIZ™ dye Size Standard (Applied Biosystems). Genotypes were scored manually using GeneMarker Software version 2.6.4 (SoftGenetics).

### Clonal assignment

We used GenoDive v. 3.06^53^ to determine the number of genets and associated ramets in each population. For *V. pompona*, the dataset included 146 individuals and 10 of 11 loci (mVplCIR016 was excluded due to missing data). For *V. odorata*, the dataset comprised 75 individuals and all 10 loci. A Stepwise Mutation Model was applied in both analyses, excluding missing data from the counts. For *V. pompona*, the minimum genetic distance threshold for distinguishing different clonal lineages was established at 1, whereas for *V. odorata*, the threshold was set at 0^54^ and the “Make clones specific to every population” option was selected for the analyses in both species. These thresholds were estimated from a frequency distribution of genetic distances, assuming that the first peak in the histogram was likely due to somatic mutations or genotyping errors^55^. The threshold was chosen between the first peak and the next increase in genetic distance frequencies. To assess clonal structure, we tested the probability of observing the detected clonal diversity by selecting the number of genotypes as the statistic for both species. We conducted 9,999 permutations, with randomization set to “alleles over individuals within populations” for *V. pompona* and “randomize alleles over individuals across all populations” for *V. odorata*.

We estimated the number of genets and calculated the clonality percentage by dividing the number of genets by the total number of individuals in each population. We also calculated Simpson’s Diversity Index (SDI), which measures genetic diversity by quantifying the probability that two randomly selected individuals belong to different genotypes. Additionally, we assessed evenness (E), which indicates the uniformity of genotype distribution among sub-populations. An evenness value of 1 indicates that all genotypes are equally represented, implying balanced diversity, whereas lower values indicate an uneven distribution, with some genotypes dominating. All these estimates were calculated for each population using GenoDive v. 3.06^53^. The analysis involved a bootstrap test for differences in clonal diversity with 9999 permutations and a jackknife procedure with increasing sample sizes, using 1000 permutations.

### Genetic diversity and structure

Genetic variation and its distribution within populations of *V. pompona* and *V. odorata* were described by estimating the average number of alleles (A), allelic richness to compensate for differences in sampling size per population (Ar), effective number of alleles (Ae), average observed (H_O_) and expected (He) heterozygosity, and the inbreeding coefficient (F_IS_). We tested each locus for significant deviations from Hardy-Weinberg equilibrium (HWE) using the exact test implemented in the Genepop software^56^. We estimated the significance of inbreeding coefficients using 1000 bootstrap samples via the *mmod* package^57^. To evaluate differences in allele frequencies among populations, we calculated the Nei’s G_ST_ index^58^. Isolation-by-distance (IBD) was measured by comparing Euclidean distances between populations using Nei’s G_ST_, and the significance of IBD was determined using a Mantel test with 9,999 permutations. All genetic diversity and structure estimates were calculated using the *adegenet*^59^ and *poppr*^54,60^ packages in R version 4.2.3^61^. All genetic diversity estimates were calculated in two separate analyses, one including clones (IC) and a second analysis excluding clones (EC) within each population (see sub-section Clonal assignment).

We visualized the dissimilarity among individuals and populations using a Discriminant Analysis of Principal Components (DAPC) as implemented in the *adegenet* package^59^. Cross-validation was conducted by partitioning the data into a training set (75%) and a validation set (25%), repeating this process 1,000 times at each step of Principal Component (PC) retention. To determine the optimal number of PC axes, we used the *xvalDapc()* function from the *adegenet* package with 30 repetitions. We then refined our search to the five PC axes closest to the previously selected PC, increasing the number of replicates to 999. We retained 25 components and three discriminant functions for *V. pompona* DAPC, while in *V. odorata* we retained seven components and three discriminant functions.

### Fine scale spatial genetic structure (FSGS)

Autocorrelation analyses based on multilocus pairwise kinship coefficients^47^ were calculated using SpaGeDi 1.5^62^. We selected distance classes with at least a minimum of 30 pairwise comparisons per distance class^63^. Fine-scale spatial genetic structure (FSGS) analyses were conducted globally for each species, combining all sampled populations into a single analysis per species. These analyses were conducted both with clones included (IC) and excluded (EC), which resulted in different distance intervals for each set of analyses. In *V. odorata*, the IC dataset had 19 distance classes, while the EC dataset had seven. For *V. pompona*, 33 distance classes were defined in the IC dataset, compared to 15 in the EC dataset. We also ran separate FSGS analyses on each population to see if patterns were consistent across populations.

Using the IC datasets, we calculated the coancestry coefficient ρ_ij_ to measure FSGS within populations that had at least 15 individuals^47^. For *V. pompona*, individual analyses were performed on six populations (VT, STH, La Pavona, Los Chocuacos, Clavera, and San Rafael), while for *V. odorata*, only three populations (PST, VT, and PPT) were analyzed. Permutation tests were conducted to evaluate the statistical significance of the observed patterns, with 10,000 permutations assigned to each test.

## Electronic supplementary material

Below is the link to the electronic supplementary material.

Supplementary Material 1

## Data Availability

The SSR genotypes are available in the Zenodo repository at https://zenodo.org/records/14195783.
