# Supplementary material for: Contrasting clonal and population genetic structure in two endangered Costa Rican Vanilla species of commercial interest
Source: Sci Rep. 2025 Jun 2;15:19295. doi: 10.1038/s41598-025-04101-5 (PMC12130201; doi:10.1038/s41598-025-04101-5)
Supplement: Supplementary file 1 — Supplementary Material 1 [file 41598_2025_4101_MOESM1_ESM.pdf]

Contrasting clonal and population genetic structure in two endangered Costa Rican *Vanilla* species of commercial interest

Maria Alejandra Serna-Sánchez[1,2*], Adam P. Karremans[1,3], Diego Bogarín[1,3,4], Eric J. Fuchs[2,3,5]

[1] Centro de Investigación Jardín Botánico Lankester, Universidad de Costa Rica, P.O. Box 302-7050, Cartago, Costa Rica

[2] Programa de Posgrado en Biología, Sistema de Estudios de Posgrado, Universidad de Costa Rica. San Pedro, San José, 11501-2060, Costa Rica

[3] Escuela de Biología, Universidad de Costa Rica, Ciudad Universitaria Rodrigo Facio, San Pedro Montes de Oca 11501-2060, Costa Rica

[4] Evolutionary Ecology Group, Naturalis Biodiversity Center, Leiden, The Netherlands.

[5] Laboratorio binacional UNAM-UCR. Morelia, Michoacán, México.

*Corresponding author: maria.serna@ucr.ac.cr

Supplementary tables S1, S2 and supplementary figures S1, S2, S3, S4, S5, S6, S7 and S8.

Supplementary Material

Supplementary Table S1. Representative voucher specimens for each of the *Vanilla pompona* and *Vanilla odorata* populations sampled in Costa Rica. Vouchers are deposited as living plants or liquid specimens at the Centro de Investigación Jardín Botánico Lankester (JBL), Cartago, Costa Rica.

Species	Population	Voucher
Vanilla pompona	STH	Karremans 6438 (living)
	VT	Karremans 7259 (living)
	PST	Karremans 7259 (living)
	Pavona	Karremans 9341 (JBL-M0190)
	Garabito	Karremans 9510 (living)
	El Hacha	Karremans 9508 (living)
	Los Chocuacos	Karremans 9516 (JBL-M0191)
	Clavera	Karremans 7044 (living); Karremans 9515 (living)
	Rio Jaris	Karremans 9656 (JBL-M0192)
	San Rafael	Karremans 9656 (JBL-M0192)
Vanilla odorata	PST	Karremans 6440 (JBL-J0618; JBL-J0920)
	VT	Karremans 9339 (JBL-J11516; JBL-K0334)
	PPT	Karremans 7255 (JBL-M0043)
	Miramar	Karremans 9646 (living); Karremans 9647 (living)
	Tigre	Karremans 9652 (living)
	Matapalo	Karremans 6439 (living)

Supplementary Table S2. Microsatellites and PCR conditions used for *V. odorata* and *V. pompona* analyses. In the SSR name, Vhu and Vro correspond to markers developed by Gigant et al (2012); Vpl were designed by Bory et al (2008). Number of individuals tested (N), allele size range (Sr) and Number of alleles (N_a). In the PCR conditions column, TD (Touchdown PCR) indicates a gradual decrease in annealing temperature over cycles to enhance specificity.

***Vanilla odorata* (N = 75)**					***Vanilla pompona* (N = 146)**				
**Mix/ Simplex**	**SSR/ Fluorophore**	**Sr**	**N_a**	**PCR conditions**	**Mix/ Simplex**	**SSR**	**Sr**	**N_a**	**PCR conditions**
1	mVhuCIR08 (FAM)	220-232	4	95°C, 15 min; 35x (94°C, 30 s; 53°C, 90 s; 72°C, 90 s); 72°C, 10 min	1	mVhuCIR07 (FAM)	212-244	15	95°C, 15 min; 35x (94°C, 30 s; 53°C, 90 s; 72°C, 90 s); 72°C, 10 min
	mVplCIR025 (TAMRA)	228-234	4			mVhuCIR08 (TAMRA)	219-247	11	
						mVhuCIR10 (HEX)	196-208	4	
2	mVplCIR005 (FAM)	234-270	4	95°C, 15 min; 35x (94°C, 30 s; 53°C, 90 s; 72°C, 90 s); 72°C, 10 min	2	mVplCIR016 (FAM)	314-346	7	95°C, 15 min; 35x (94°C, 30 s; 50°C, 90 s; 72°C, 90 s); 72°C, 10 min
	mVplCIR026 (TAMRA)	212-228	5			mVplCIR019 (HEX)	204-218	3	
3	mVroCIR01 (FAM)	120-148	4	TD: 95°C, 15 min; 10x (94°C, 30 s; 63-55°C, 45 s; 72°C, 90 s); 24x (94°C, 30 s; 55°C, 90 s; 72°C, 90 s); 72°C, 10 min	3	mVroCIR01 (FAM)	132-136	3	TD: 95°C, 15 min; 10x (94°C, 30 s; 63-55°C, 45 s; 72°C, 90 s); 24x (94°C, 30 s; 55°C, 90 s; 72°C, 90 s); 72°C, 10 min
	mVroCIR05 (TAMRA)	196-202	3			mVroCIR05 (TAMRA)	196-202	4	
	mVplCIR047 (HEX)	305-307	2						
Simplex	mVhuCIR07 (TAMRA)	200-264	6	95°C, 3 min; 35x (95°C, 30 s; 50°C, 45 s; 72°C, 1 min); 72°C, 5 min	Simplex	mVhuCIR11 (HEX)	238-242	3	94°C, 4 min; 40x (94°C, 1 min; 57°C, 30 s; 72°C, 1min); 72°C, 7min
	mVroCIR03 (FAM)	294-348	4	95°C, 3 min; 35x (95°C, 30 s; 53°C, 45 s; 72°C, 90 s); 72°C, 5 min		mVplCIR015 (FAM)	258-278	3	95°C, 3 min; 35x (95°C, 30 s; 55°C, 45 s; 72°C, 1 min); 72°C, 5 min
	mVplCIR031 (TAMRA)	344-348	3	94°C, 4 min; 35x (94°C, 1 min; 54°C, 30 s; 72°C, 1 min); 72°C, 7 min		mVplCIR026 (HEX)	212-218	4	95°C, 3 min; 35x (95°C, 30 s; 53°C, 45 s; 72°C, 90 s); 72°C, 5 min
						mVplCIR031 (TAMRA)	342-348	3	94°C, 4 min; 35x (94°C, 1 min; 54°C, 30 s; 72°C, 1 min); 72°C, 7 min

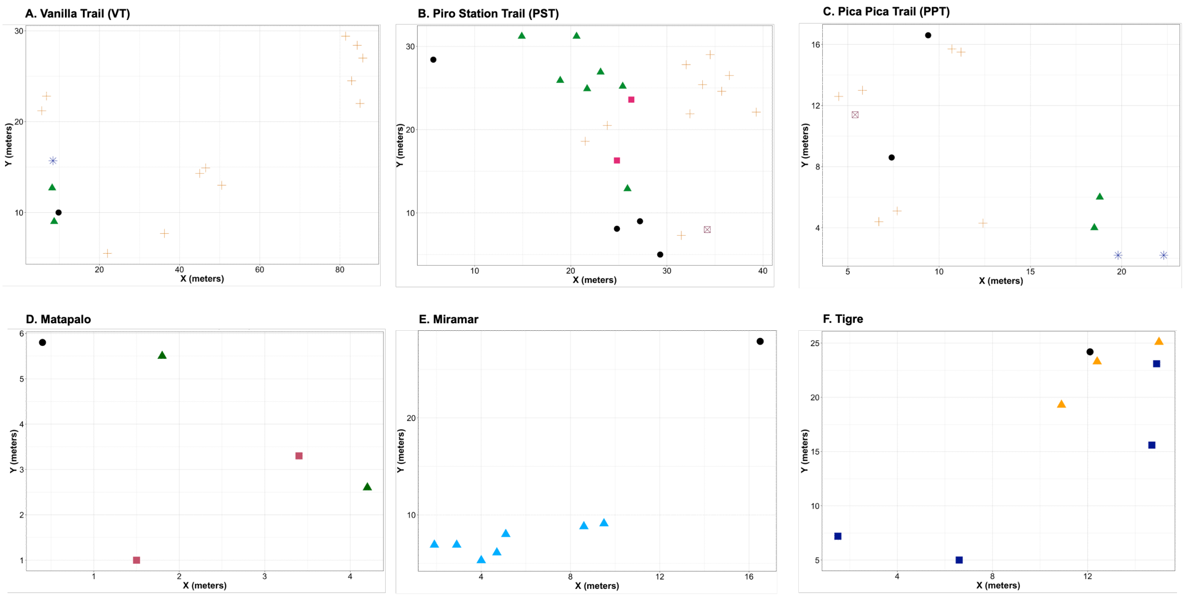

Supplementary Figure S1. Spatial distribution of *Vanilla odorata* individuals across different populations, with clonal groups represented in the same color, and unique genets represented with black dots.

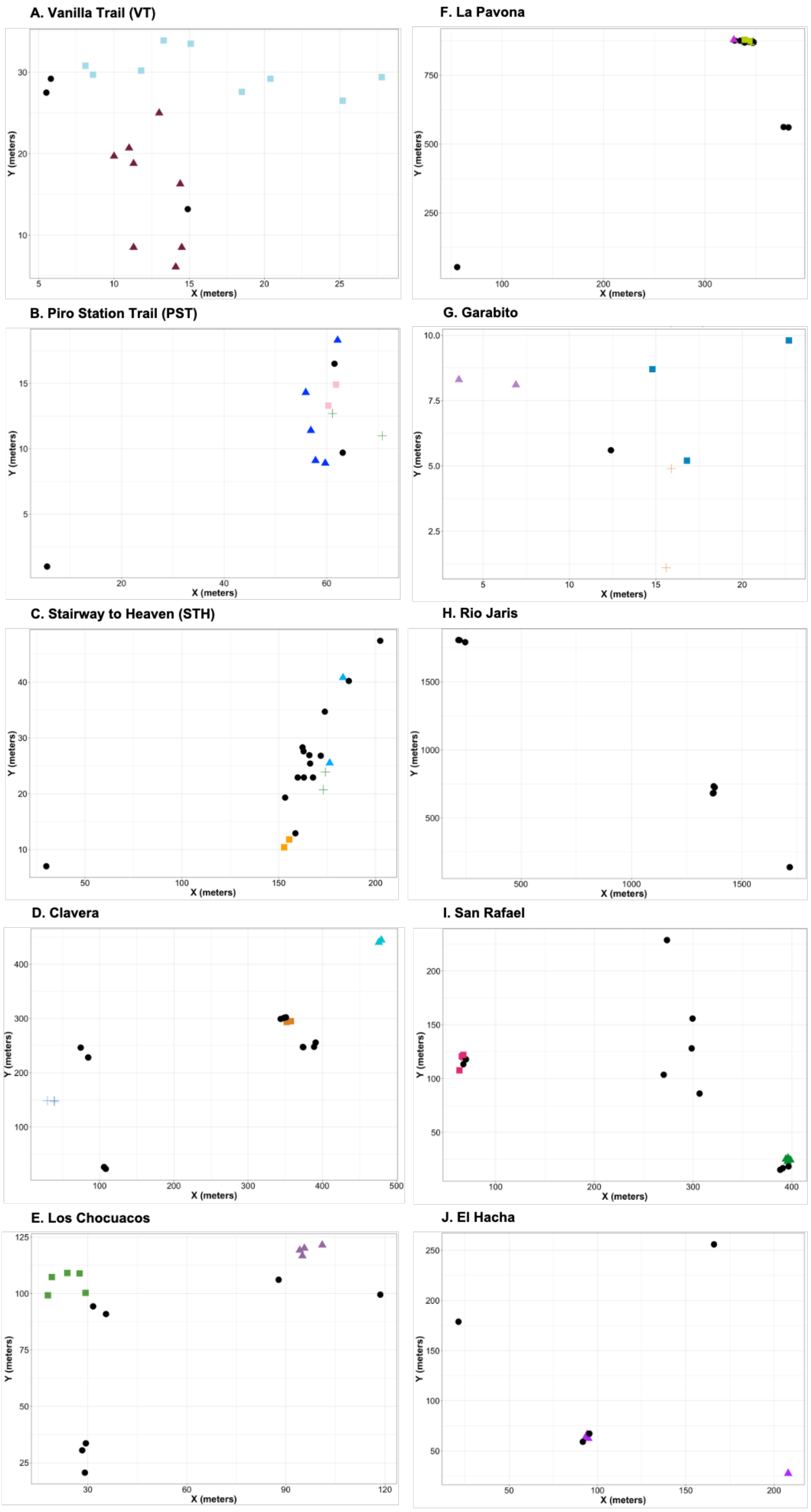

Supplementary Figure S2. Spatial distribution of *V. pompona* individuals across different populations, with clonal groups represented in the same color, and unique genets represented with black dots.

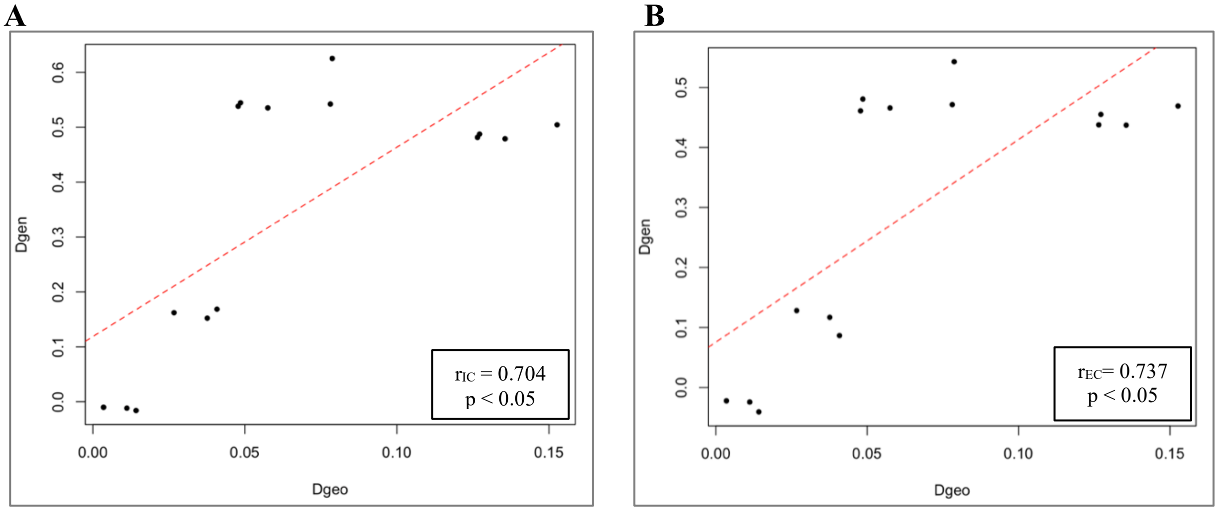

Supplementary Figure S3. Isolation-by-distance (IBD) graph with Nei's genetic distance plotted against pairwise Euclidian geographical distance for *V. odorata* **A.** Including clones (r_{IC}), **B.** Excluding clones (r_{EC})

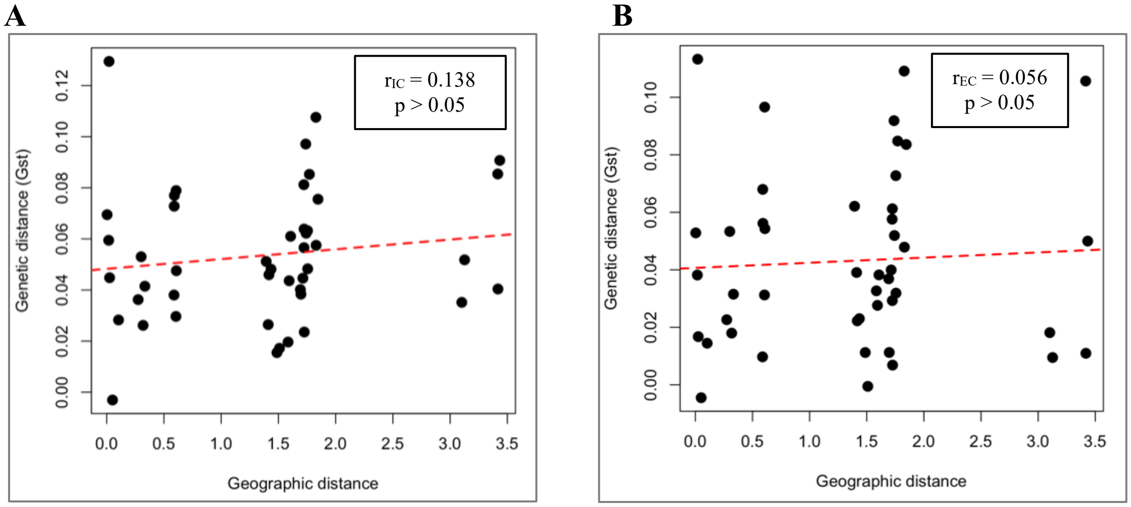

Supplementary Figure S4. Isolation-by-distance (IBD) graph with Nei's genetic distance plotted against pairwise Euclidian geographical distance for *V. pompona* – population level. **A.** Including clones (r_{IC}), **B.** Excluding clones (r_{EC})

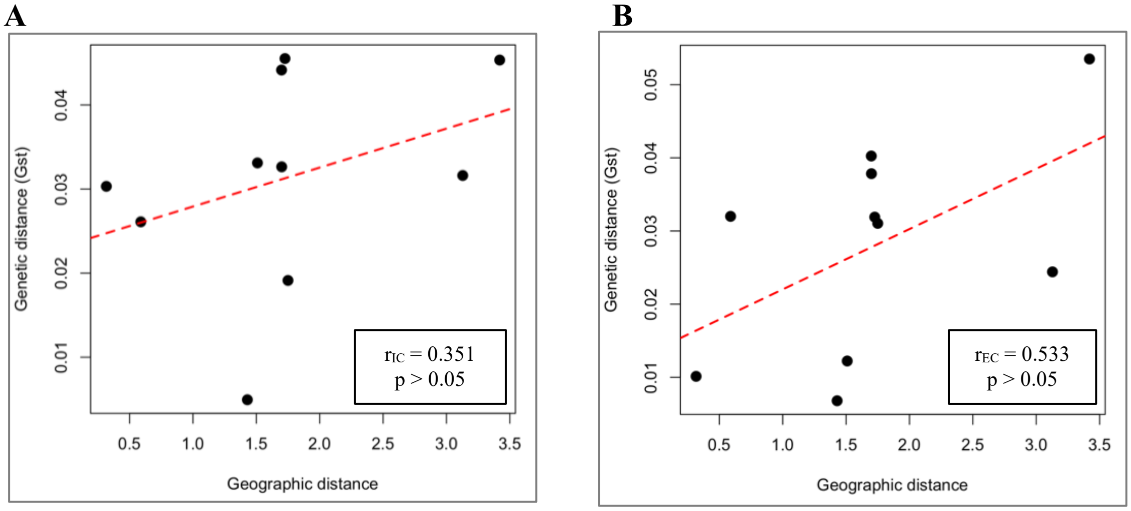

Figure S5. Isolation-by-distance (IBD) graph with Nei's genetic distance plotted against pairwise Euclidian geographical distance for *V. pompona* – regional level. **A.** Including clones (r_{IC}), **B.** Excluding clones (r_{EC})

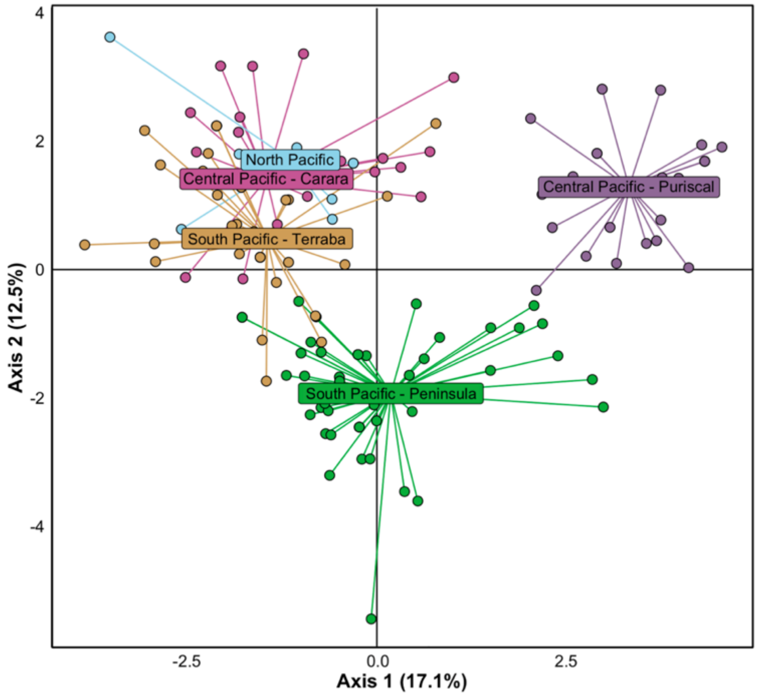

Figure S6. Discriminant analysis of principal components (DAPC) on *V. pompona* including clones (IC), showing individuals clustered into five regions.

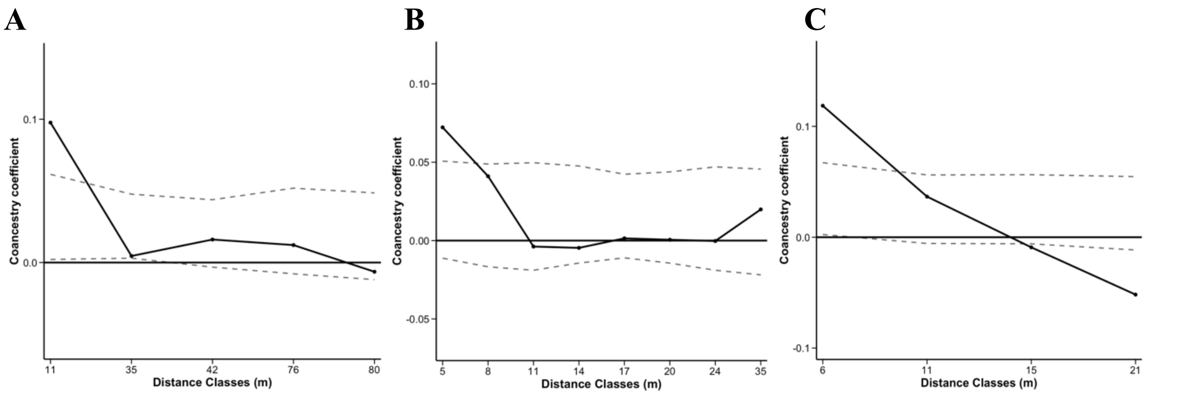

Figure S7. Autocorrelogram of estimated coancestry based on Loiselle coefficient (Loiselle et al., 1995) for *V. odorata* populations with the dataset including clones. Dashed lines represent lower and upper 95% confidence limits around zero relationship. **A.** VT, **B.** PST, **C.** PPT

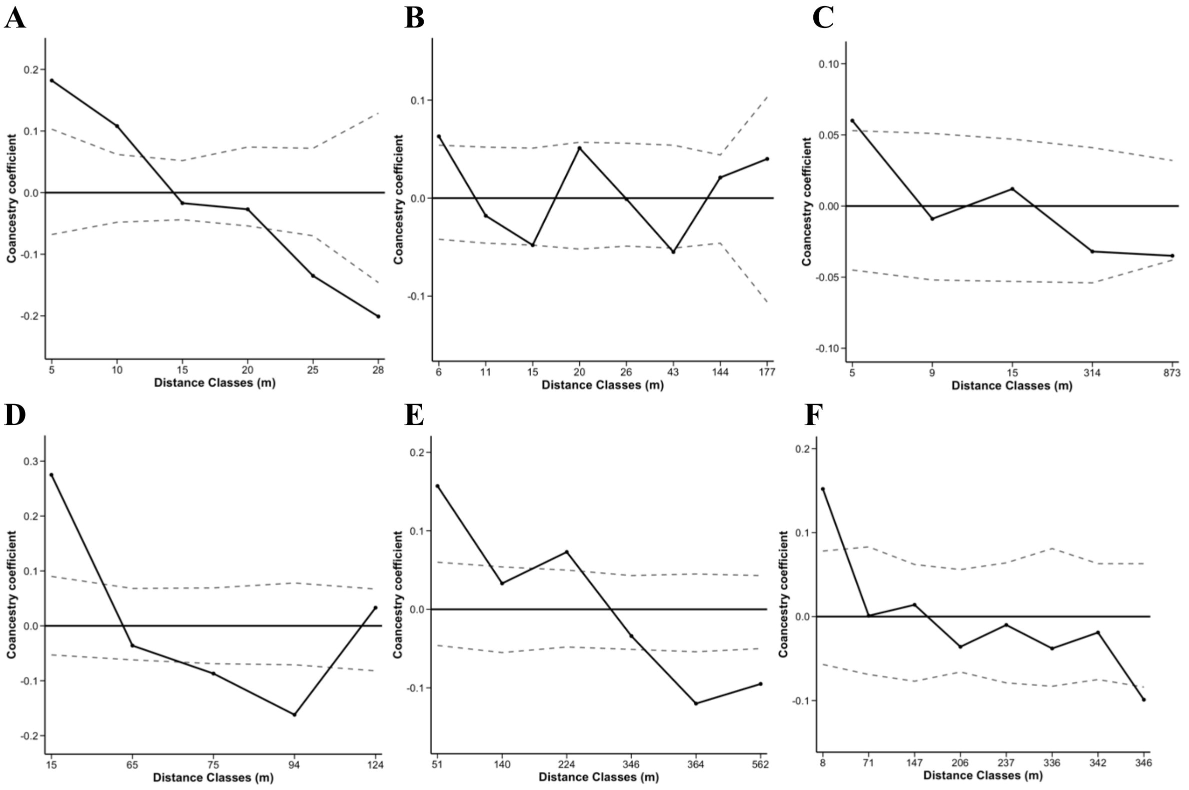

Figure S8. Autocorrelogram of estimated coancestry based on Loiselle coefficient (Loiselle et al., 1995) for *V. pompona* populations with the dataset including clones. Dashed lines represent lower and upper 95% confidence limits around zero relationship. **A.** VT, **B.** STH, **C.** La Pavona, **D.** Los Chocuacos, **E.** Clavera, **F.** San Rafael
